# D-Dimer Concentrations and COVID-19 Severity: A Systematic Review and Meta-Analysis

**DOI:** 10.3389/fpubh.2020.00432

**Published:** 2020-08-04

**Authors:** Panagiotis Paliogiannis, Arduino Aleksander Mangoni, Paola Dettori, Gheyath K. Nasrallah, Gianfranco Pintus, Angelo Zinellu

**Affiliations:** ^1^Department of Medical, Surgical and Experimental Sciences, University of Sassari, Sassari, Italy; ^2^Department of Clinical Pharmacology, College of Medicine and Public Health, Flinders University, Adelaide, SA, Australia; ^3^Cure and Health Center, Sassari, Italy; ^4^Department of Biomedical Sciences, College of Health Sciences, Qatar University, Doha, Qatar; ^5^Biomedical Research Centre, Qatar University, Doha, Qatar; ^6^Department of Medical Laboratory Sciences, College of Health Sciences, and Sharjah Institute for Medical Research, University of Sharjah, Sharjah, United Arab Emirates; ^7^Biomedical Sciences, University of Sassari, Sassari, Italy

**Keywords:** D-dimer, coagulation, thrombosis, COVID-19, SARS-CoV-2

## Abstract

Coronavirus disease 2019 (COVID-19) is a recently described infectious disease caused by severe acute respiratory syndrome coronavirus 2 (SARS-CoV-2). Since late 2019, COVID-19 has rapidly spread in virtually all countries, imposing the adoption of significant lockdown and social distancing measures. The activation of the coagulation cascade is a common feature of disseminated intravascular coagulation and adverse clinical outcomes in COVID-19 patients. In this study, we conducted a meta-analysis aiming to investigate differences in serum D-dimer concentrations in patients with and without severe COVID-19 disease. An electronic search in Medline (PubMed), Scopus and Web of Science was performed with no language restrictions, and 13 articles were reporting on 1,807 patients (585, 32.4% with severe disease) were finally identified and included in the meta-analysis. The pooled results of all studies revealed that the D-dimer concentrations were significantly higher in patients with more severe COVID-19 (SMD: 0.91 mg/L; 95% CI, 0.75 to 1.07 mg/L, *p* < 0.0001). The heterogeneity was moderate (*I*^2^ = 46.5%; *p* = 0.033). Sensitivity analysis showed that the effect size was not modified when any single study was in turn removed (effect size range, 0.87 mg/L to 0.93 mg/L). The Begg's (*p* = 0.76) and Egger's tests (*p* = 0.38) showed no publication bias. In conclusion, our systematic review and meta-analysis showed that serum D-dimer concentrations in patients with severe COVID-19 are significantly higher when compared to those with non-severe forms.

## Introduction

Coronavirus disease 2019 (COVID-19) is a recently described infectious disease caused by severe acute respiratory syndrome coronavirus 2 (SARS-CoV-2) (1). Since late 2019, COVID-19 has rapidly spread in virtually all countries, affecting more than two million people and causing more than 150,000 deaths worldwide (data from 17 Apr 2020, https://www.worldometers.info/coronavirus/). These figures are continuously growing despite the adoption of significant lockdown and social distancing measures, particularly in Eastern Asia, Europe, and North America (2). The rapid expansion and the relatively high lethality may depend on several biological characteristics, such as the high infectivity of SARS-CoV-2, the high percentage of asymptomatic vectors, and the relatively long incubation period (3). However, significant knowledge gaps remain in the pathophysiology of the disease. In this context, a better knowledge of the factors that are responsible for the development of significant clinical complications in a subgroup of COVID-19 patients, indicating high disease severity, might lead to the identification of better pharmacological and non-pharmacological therapies and care pathways. This would improve patient outcomes, and reduce the current burden on health care systems, pending the development of effective vaccines. There is increasing evidence that SARS-CoV-2 induces, in severe cases, a cytokine storm that triggers the coagulation cascade, causing thrombotic complications (4). This is clinically relevant as the activation of the coagulation cascade is a common feature of disseminated intravascular coagulation (DIC) and adverse clinical outcomes in COVID-19 patients and appears to be more frequent than what observed in patients suffering from severe forms of SARS-CoV in 2003 (5). The key pathophysiological role of DIC in the clinical progress of COVID-19 is further supported by the presence, in autopsies of patients succumbing to the disease, of fibrinous thrombi, endothelial tumefaction, and megakaryocytes in small pulmonary arteries and pulmonary capillaries (6).

The D-dimer, a fibrin degradation product, is a relatively small protein fragment that is present in the blood following degradation of blood clots by fibrinolysis. The determination of circulating D-dimer concentrations is a sensitive test in clinical practice to diagnose thrombotic states, including pulmonary embolism and DIC (7). Therefore, elevations in D-dimer levels in COVID-19 patients might be helpful to rapidly identify those that have high disease severity, pulmonary complications, and risk of venous thromboembolism in the setting of a pro-thrombotic state. This would assist with risk stratification and the early introduction of therapeutic measures that might reduce COVID-19 related morbidity and mortality.

A recent meta-analysis has shown that patients with severe forms of COVID-19 have higher D-dimer concentrations when compared to those with milder forms (8). However, only a small number of studies in a total of 553 patients were selected. Furthermore, in this meta-analysis the heterogeneity across the studies was extremely high, *I*^2^ 94%, *P* < 0.001). Therefore, we conducted an updated meta-analysis that takes into account additional studies to investigate differences in serum D-dimer concentrations in patients with and without severe COVID-19 disease.

## Materials and Methods

### Study Search and Selection

An electronic search in Medline (PubMed interface), Scopus, and Web of Science was performed using the keywords “D-dimer” AND “coronavirus” OR “D-dimer” AND “COVID-19.” The inclusion criteria were: (a) studies reporting continuous data on serum D-dimer concentrations in COVID-19 patients, (b) articles dividing COVID-19 patients in severity classes, (c) articles including adult patients, (d) studies approved by an ethical committee, and (e) articles published from 1st January 2020 to the date of the electronic search (14th April, 2020). There were no language restrictions. The titles, abstracts and full texts of the publications retrieved were screened by two independent investigators (PP and AZ). The reference list of the studies identified was also checked in order to identify additional studies. The Newcastle—Ottava Scale (NOS) was used for quality assesment. This meta-analysis was conducted according to the Preferred Reporting Items for Systematic Reviews and Meta-Analyses (PRISMA) guidelines (Supplementary Materials).

### Statistical Analysis

Standardized mean differences (SMD) were used to build forest plots of continuous data and to evaluate differences in serum D-dimer concentrations between severe and non-severe patients with COVID-19 disease. A *P*-value < 0.05 was considered statistically significant, and 95% confidence intervals (CIs) were reported. When necessary, the mean and standard deviation values were extrapolated from median and IQR values, as previously reported by Wan et al. (9). Heterogeneity of SMD across studies was tested using the Q statistic (significance level at *p* < 0.10). The *I*^2^ statistic, a quantitative measure of inconsistency across studies, was also calculated (*I*^2^ < 25%, no heterogeneity; *I*^2^ between 25 and 50%, moderate heterogeneity; *I*^2^ between 50 and 75%, large heterogeneity; and *I*^2^ > 75%, extreme heterogeneity) (10, 11). In analyses in which heterogeneity was high, a random-effects model was applied. To investigate the influence of an individual study on the overall risk estimate, a sensitivity analysis was conducted by sequentially excluding one study at a time (12). Begg's adjusted rank correlation test and Egger's regression asymmetry test, for the analysis of associations between study size and magnitude of effect were used to evaluate the presence of potential publication bias (13, 14). The Duval and Tweedie “trim and fill” procedure to identify and correct for funnel plot asymmetry arising from publication bias was also used (15). Statistical analyses were performed using Stata 14 (STATA Corp., College Station, TX, USA).

## Results

### Study Selection Results and Characteristics

**Figure 1:**
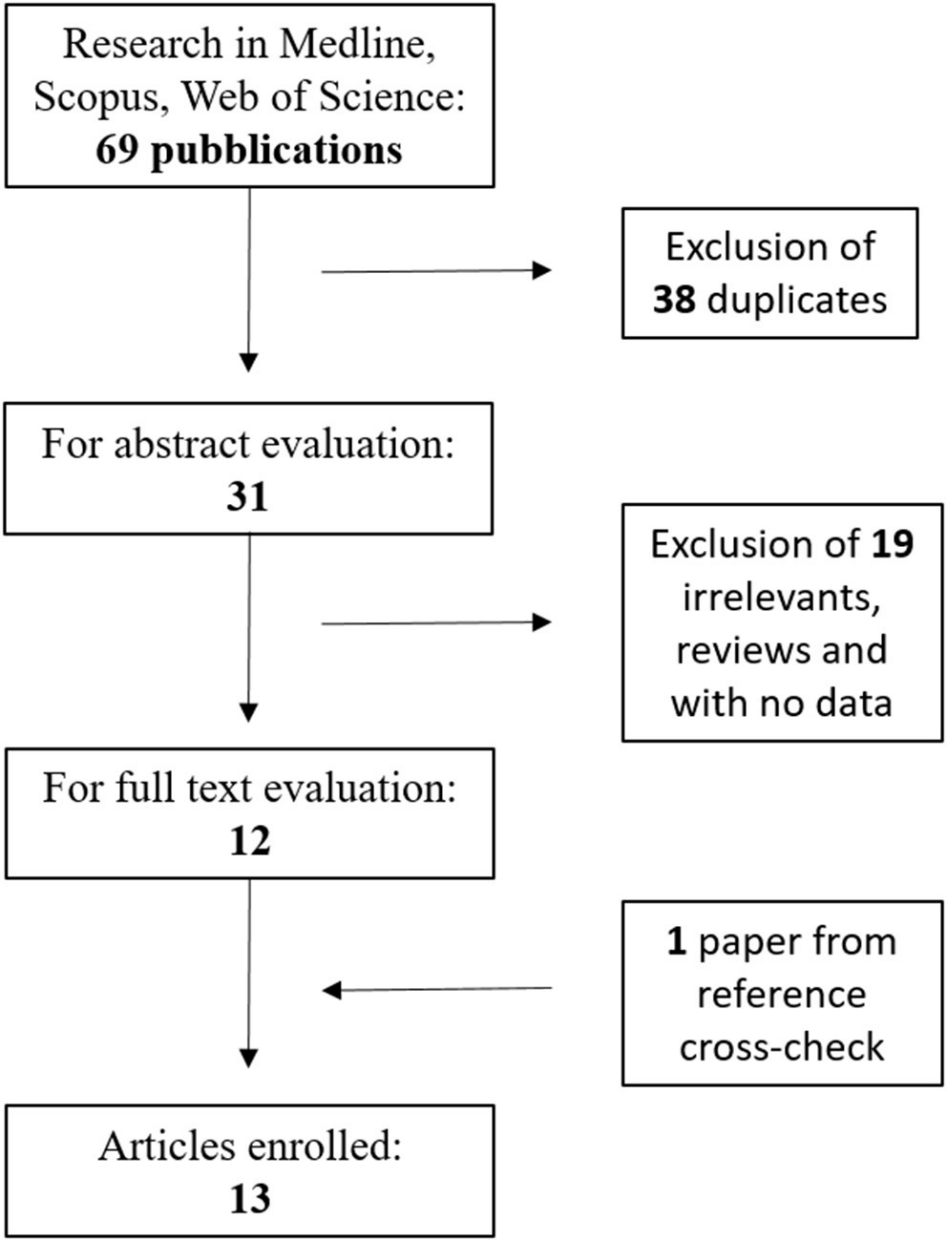
Flow-chart illustrating the electronic search strategy and results.

The flow diagram of the literature search performed is presented in Figure 1. From an initial total of 69 studies, 13 were finally identified and included in the meta-analysis (16–28); the total number of COVID-19 patients in these studies was 1,807. Among them, 585 (32.4%) were affected by a severe form of COVID-19 (Table 1). The NOS quality assessment is described in Table 1.

**Table 1:** Characteristics of the patients and D-dimer values in the studies enrolled for meta-analysis.

**References**	**NOS stars**	**Total cases (severe)**	**Age**	**Males, *n* (%)**	**D-dimer total (mg/L)**	**D-dimer severe (mg/L)**	**D-dimer non-severe (mg/L)**
Zhou et al. (16)	6	17 (5)	NA	6 (35)	NA	0.28 ± 0.11	0.29 ± 0.11
Tang et al. (17)	7	449 (134)	65 (mean)	268	1.94 (0.90–9.44)	4.70 (1.42–21.00)	1.47 (0.78–4.16)
Chen et al. (18)	6	21 (11)	56 (median)	17 (81)	0.5 (0.4–1.8)	2.6 (0.6–18.7)	0.3 (0.3–0.4)
Chen et al. (19)	6	274 (113)	62 (median)	171 (62)	1.1 (0.5–3.2)	4.6 (1.3–21.0)	0.6 (0.3–1.3)
Wan et al. (20)	6	135 (40)	47 (median)	72 (53)	0.4 (0.2–0.6)	0.6 (0.4–1.1)	0.3 (0.2–0.5)
Gao et al. (21)	6	43 (15)	45 (mean)	26 (58)	NA	0.49 (0.29-0.91)	0.21 (0.19–0.27)
Han et al. (22)	6	84 (35)	NA	NA	NA	19.11 ± 35.48	2.14 ± 2.88
Zhou et al. (23)	7	191 (54)	56 (median)	119 (62)	0.8 (0.4–3.2)	5.2 (1.5–21.1)	0.6 (0.3–1.0)
Wu et al. (24)	6	201 (84)	51 (median)	128 (64)	0.61 (0.35–1.28)	1.16 (0.46–5.37)	0.52 (0.33–0.93)
Liu et al. (25)	6	30 (4)	35 (mean)	10 (33)	NA	1.54 ± 1.22	0.26 ± 0.08
Zhang et al. (26)	7	138 (56)	57 (median)	71 (51)	0.2 (0.1–0.5)	0.4 (0.2–2.4)	0.2 (0.1–0.3)
Tang et al. (27)	7	183 (21)	54 (mean)	98 (53)	0.66 (0.38–1.50)	2.12 (0.77–5.27)	0.61 (0.35–1.29)
Huang et al. (28)	7	41 (13)	49 (median)	30 (73)	0.5 (0.3–1.3)	2.4 (0.6–14.4)	0.5 (0.3–0.8)
Total		1,807 (585)					

*NOS, Newcastle – Ottawa Scale; NA, not available*.

All selected studies were conducted in China. Six articles defined severe cases based on current clinical guidelines (20–22, 24–26), four defined severe cases as those who died in comparison to survivors (17, 19, 23, 27), and three had alternative definitions (disease progression vs. no progression or admission vs. no admission to intensive care units) (16, 18, 28).

### Meta-Analysis

**Figure 2:**
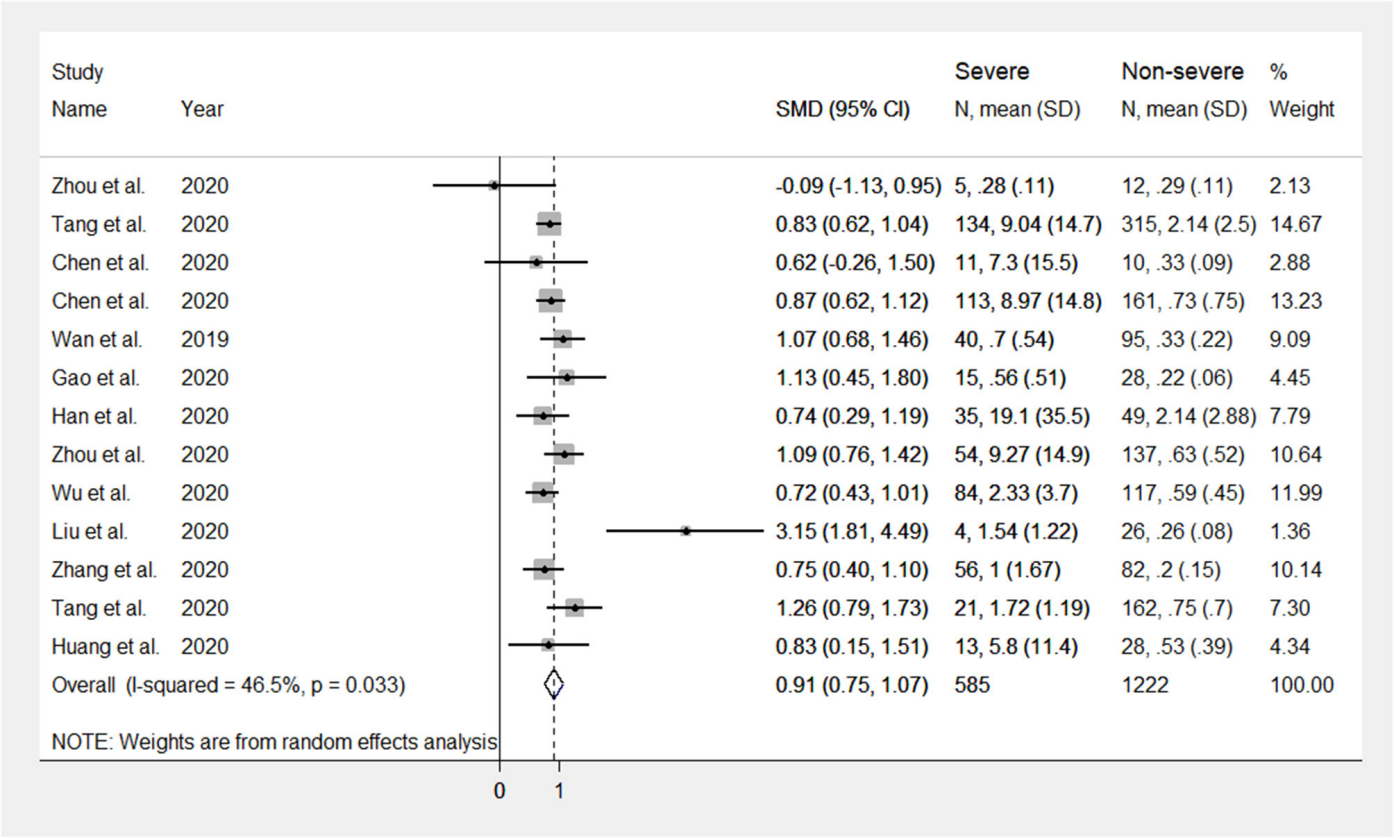
Forest plot illustrating D-dimer standardized mean differences (SMD) in patients with and without severe COVID-19.

**Figure 3:**
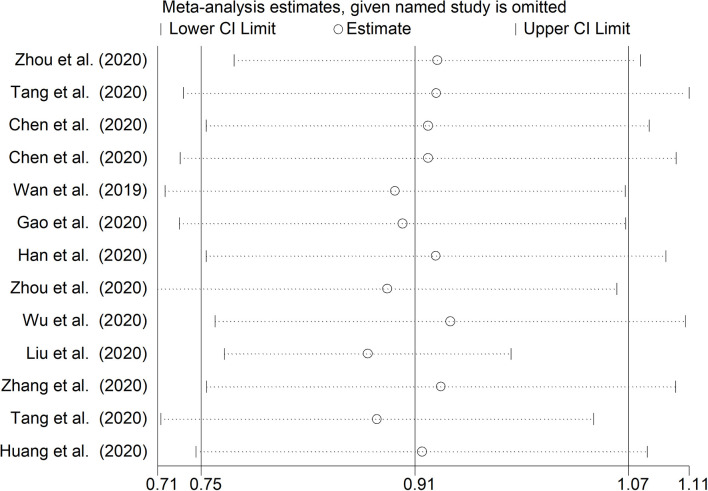
Sensitivity analysis of the studies enrolled. CI, confidence interval.

**Figure 4:**
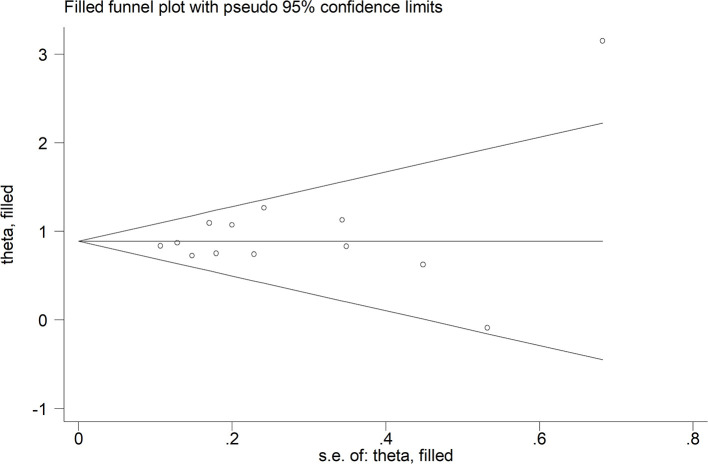
Trim-and-fill analysis of the studies enrolled.

**Figure 5:**
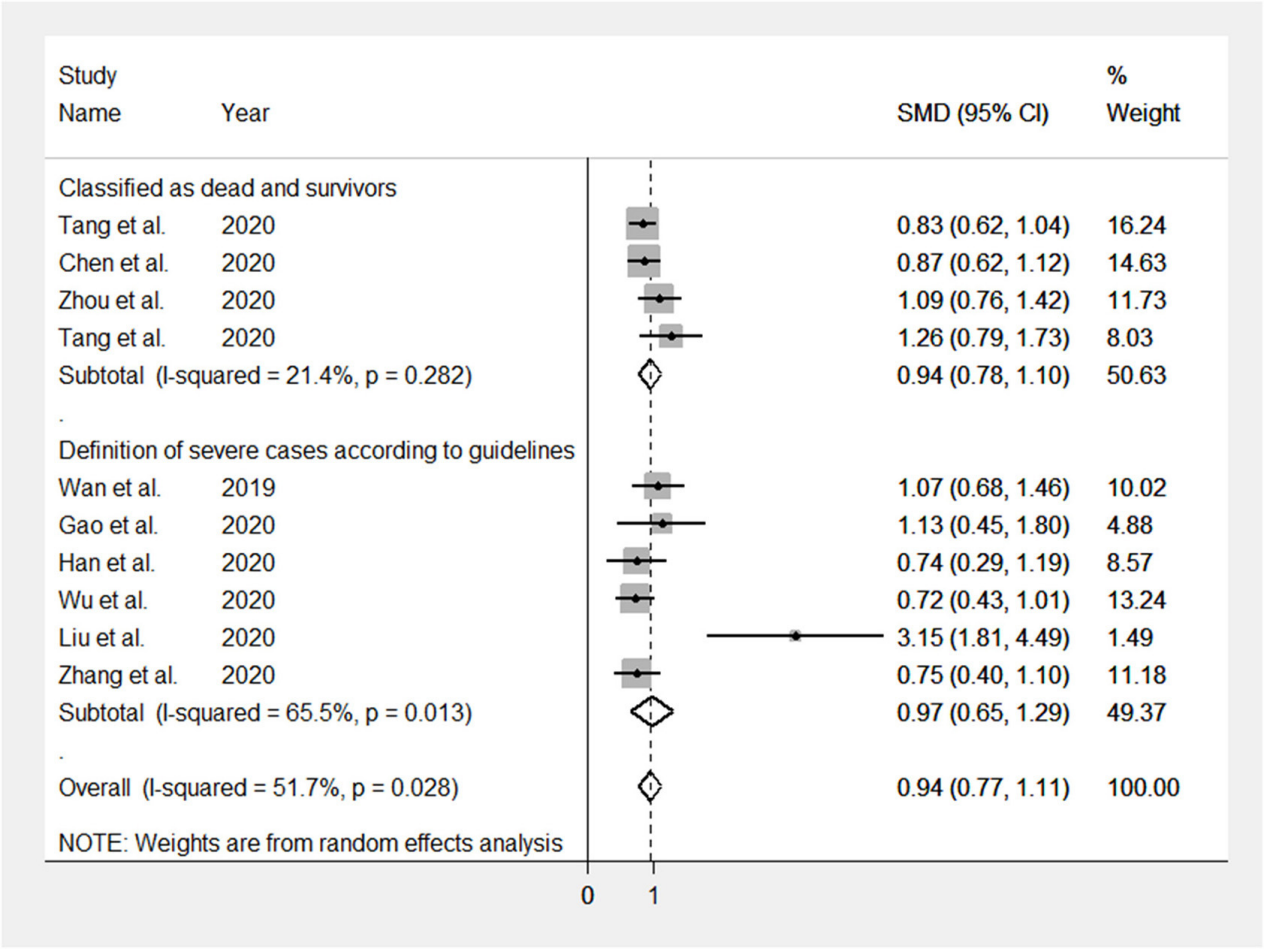
Forest plots illustrating subgroup analyses on the basis of the definition of COVID-19 severity. SMD: standardized mean difference.

The mean differences in serum D-dimer concentrations between COVID-19 patients with or without severe disease in the 13 studies are shown in Figure 2. In 12 studies, patients with severe COVID-19 displayed higher D-dimer serum concentrations when compared to those with milder forms (mean difference range, 0.62–3.15 mg/L) (17–28). By contrast, in the remaining study, the D-dimer concentration was found to be mildly higher in patients with non-severe forms of COVID-19 (mean difference 0.09 mg/L) (16). The pooled results of all studies revealed that the D-dimer concentrations were significantly higher in patients with more severe COVID-19 (SMD: 0.91 mg/L; 95% CI, 0.75 to 1.07 mg/L, *p* < 0.0001). The heterogeneity was moderate (*I*^2^ = 46.5%; *p* = 0.033). Sensitivity analysis showed that the effect size was not modified when any single study was in turn removed (effect size range, 0.87 mg/L−0.93 mg/L, Figure 3). The Begg's (*p* = 0.76) and Egger's tests (*p* = 0.38) showed no publication bias. Accordingly, the trim-and-fill analysis found that no study was missing or should be added (Figure 4). In meta-regression analysis, no correlation was found either between SMD and age (*p* = 0.37) or between SMD and gender (*p* = 0.41). Notably, the age ratio between patients with more severe COVID-19 and those with milder forms was relatively small, between 1.0 and 1.3, in all studies. In addition, as reported in Figure 5, there were no significant differences in SMD values between the subgroup of patients classified according to guidelines (SMD: 0.94 mg/L; 95% CI 0.78 to 1.10 mg/L, *p* < 0.0001) and the subgroup classified as dead or survivors (SMD: 0.97 mg/L; 95% CI 0.65 to 1.29 mg/L, *p* < 0.0001), although in the first group a significantly lower heterogeneity was observed (*I*^2^ = 21.4%, *p* < 0.28 vs. *I*^2^ = 65.5%, *p* < 0.013).

## Discussion

Our updated meta-analysis of 13 studies in 1,807 COVID-19 patients showed that the serum D-dimer concentrations in patients with severe forms of the disease were significantly higher than those in patients with milder forms. When compared to a recent meta-analysis of four studies in a total of 553 COVID-19 patients, the observed SMD values were relatively small, 0.91 mg/L (3, 17, 27, 28). Furthermore, in our meta-analysis the heterogeneity was substantially lower, *I*^2^ 46.5 vs. 94% (8). These results further support the presence of a pro-thrombotic state, and possibly DIC, in COVID-19 patients with severe disease, potentially accounting for the structural and functional lung abnormalities commonly reported in this subgroup. In support of this hypothesis, recent autoptic reports have shown alterations compatible with DIC in the lungs of COVID-19 patients (6). Interestingly, we observed no significant associations between increasing SMD values and the age ratio between patients with more severe COVID-19 and those with milder forms, despite the established age-related increase in serum D-dimer concentrations (7). As patients with severe COVID-19 disease are also significantly older than subgroups with milder forms (29), our findings suggest that the reported differences in serum D-dimer concentrations are independent of age differences in patients with different disease severity. Although this further supports the presence of DIC as the primary marker of D-dimer elevations and COVID-19 severity, additional studies in cohorts with higher age ratios between patients with more severe COVID-19 and those with milder forms are required to confirm this proposition.

Pending further research to investigate the cause-effect relationship between serum D-dimer concentrations, COVID-19 disease severity, the onset of pulmonary complications and clinical outcomes, the identification of D-dimer as a biomarker of COVID-19 severity is potentially clinically relevant. Its relatively simple and inexpensive determination might assist, particularly with serial assessments, with the rapid identification of those patients developing DIC, pulmonary compromise, or at risk of venous thromboembolism that requires aggressive care and intensive monitoring (30). While the development of COVID-19 vaccines is eagerly awaited, a better understanding of the pathophysiological mechanisms responsible for the clinical deterioration and increased risk of death in affected patients is likely to be beneficial. For example, the rapid initiation of DIC therapies, instigated by high D-dimer concentrations and the presence of other diagnostic criteria, might provide additional therapeutic advantages in severe COVID-19 patients already receiving ventilatory and circulatory support (30). This proposition is supported by the findings of a recent study in 449 severe COVID-19 patients with significant elevations of serum D-dimer concentrations and/or criteria for DIC. The administration of low molecular weight heparin in these patients was associated with a significant improvement in 28-day survival when compared to non-users (27).

The moderate heterogeneity in the studies enrolled might depend on the different definitions of disease severity; in six studies, available clinical guidelines were followed, mainly the “new coronavirus pneumonia diagnosis and treatment plan” (versions 4 and 5) developed by the National Health Committee of the People's Republic of China (31). In four studies, the severity of the disease was based on survivorship or death, and finally, in the remaining three studies, further classifications were used, such as disease progression vs. no progression, and admission or no admission in intensive care units. For this reason, we performed subgroup analyses, which showed no significant differences in SMD values between the subgroup of patients classified according to clinical guidelines and the subgroup classified as dead or survivors. Further potential issues are that all the included studies were carried out in China, no strict diagnostic performance was investigated, and no specific guidelines for reporting (such as the Standards for Reporting Diagnostic Accuracy Studies, STARD recommendations) were followed in each individual study. Other potential sources of heterogeneity, not described in the identified studies, include differences in the timing of blood sample collection and analytical protocols for D-dimer measurement.

In conclusion, our systematic review and meta-analysis showed that the serum concentrations of D-dimer, a fibrin degradation product that is used to diagnose the presence of a pro-thrombotic state, are significantly higher in patients with severe COVID-19 when compared to those with non-severe forms. This suggests that D-dimer concentrations might be helpful to rapidly identify COVID-19 patients with high risk of pulmonary complications and venous thromboembolism, facilitating the early initiation of effective therapies. However, further studies are required to confirm such findings in different geographical areas, using robust assessment methods, and to investigate the associations between D-dimer concentrations, COVID-19 disease progress, response to treatment, and overall clinical prognosis.

## Data Availability Statement

All datasets generated for this study are included in the article/Supplementary Material.

## Author Contributions

Conceptualization: AZ. Methodology: PP and AM. Software and writing—original draft preparation: PP, AM, and PD. Validation: PP, AM, and AZ. Formal analysis: PP, PD, GN, and GP. Investigation and resources: AZ, PP, AM, GN, and GP. Data curation and project administration: GN and GP. Writing—review and editing and funding acquisition: GN, GP, and AZ. Visualization: PP and AM. Supervision: AZ. All authors have read and agreed to the published version of the manuscript.

## Conflict of Interest

The authors declare that the research was conducted in the absence of any commercial or financial relationships that could be construed as a potential conflict of interest.
